# Vater’s ampullary carcinoma increases the risk of clinically relevant postoperative pancreatic fistula after pancreaticoduodenectomy: A retrospective and propensity score-matched analysis

**DOI:** 10.1186/s12876-022-02128-w

**Published:** 2022-02-06

**Authors:** Yifei Yang, Xu Fu, Saisai Zhu, Zhenghua Cai, Yudong Qiu, Liang Mao

**Affiliations:** Department of Hepatopancreatobiliary Surgery, Nanjing Drum Tower Hospital, The Affiliated Hospital of Nanjing University Medical School, No. 321 Zhongshan Road, Nanjing, 210008 Jiangsu Province China

**Keywords:** Clinically relevant pancreatic fistula, Vater’s ampullary carcinoma, Pancreaticoduodenectomy

## Abstract

**Background:**

Postoperative pancreatic fistula (POPF) is a frequent complication after pancreaticoduodenectomy (PD). This study aimed to investigate the impact of Vater’s ampullary carcinoma (VAC) on clinically relevant POPF (CR-POPF) in patients undergoing PD.

**Methods:**

Clinical data were gathered retrospectively from January 2018 to December 2020 for all patients undergoing PD. The univariate and multivariate analysis were used to identify independent risk factors of CR-POPF. A propensity score-matched (PSM) analysis at a ratio of 1:1 was performed to minimize bias from baseline characteristics between VAC and non-VAC groups. Main postoperative complications were compared between the two groups after PSM.

**Results:**

In 263 patients, 94 (35.7%) patients were diagnosed as VAC. CR-POPF occurred in 99 (37.6%) patients and VAC was identified as an independent risk factor of CR-POPF in multivariate logistic regression analysis (OR = 0.548, 95% CI = 0.327–0.920, *P* = 0.023). After PSM, there were similar baseline characteristics between the VAC and non-VAC group. Moreover, VAC group had a higher rate of CR-POPF (*P* = 0.025) and intra-abdominal infection (*P* = 0.015) compared to the non-VAC group.

**Conclusions:**

In patients undergoing PD, VAC increases the risk of CR-POPF and several other postoperative complications.

## Background

Pancreaticoduodenectomy (PD) is a complex operation performed in various benign and malignant disease localized in the pancreatic head or periampullary region with high morbidity and mortality [1–3]. Clinically relevant postoperative pancreatic fistula (CR-POPF), which is the most frequent complication, sometimes triggers life-threatening complications, such as post-pancreatectomy hemorrhage (PPH), sepsis, and death. As reported previously, many factors affect the occurrence of CR-POPF, such as age, sex, body mass index (BMI), pancreatic texture, diameter of main pancreatic duct (MPD) [4–6]. Complication rate, especially of pancreatic fistula (PF) have differed greatly among pancreatic ductal adenocarcinoma (PDAC), distal cholangiocarcinoma (DCC), and Vater’s ampullary carcinoma (VAC) [7]. Several studies identified that underlying pathology type was significantly associated with the occurrence of CR-POPF as PDAC and chronic pancreatitis are always correlated with firm pancreatic texture and dilated MPD [8, 9]. However, few studies established that patients with VAC have a higher risk of postoperative complications especially CR-POPF.

In this study, we explore the risk factors of CR-POPF, analyze the incidence of postoperative complications after PD for patients with VAC and compare them with patients who underwent the same procedure for non-VAC.

## Methods

### Patients

Data were collected retrospectively for patients who had undergone PD between January 2018 and December 2020 in our center. The study was approved by the Health Research Ethics Board of Drum Tower Hospital of Nanjing University Medical School (2021–271-01). The inclusion criteria were as follows: (a)met the indication for PD; (b) no evidence of locoregional unresectable or other active cancers, and (c) > 18 years of age. The exclusion criteria were as follows: (a) undergone simultaneous hepatic or colon resection; (b) clinical data were incomplete, and (c) history of neoadjuvant chemotherapy. Patients’ demographics, preoperative laboratory tests, pathological result, and postoperative complications were all obtained.

### Surgical procedures and perioperative management

All PDs were successfully performed using a standard surgical technique. A modified Child’s method was performed in the reconstruction of the digestive tract. Pancreaticojejunostomy (PJ) was performed with a manual duct-to-mucosal, end-to-side, and double-layer interrupted anastomosis method. According to the diameter of MPD, an internal unabsorbed Wirsung duct stent was placed. Hepaticojejunostomy and gastrojejunostomy were performed on the same jejunal loop. At the end of operation, two closed-suction peritoneal drainage tubes were routinely placed at the superior, inferior sides of PJ.

A standard perioperative management was performed in all patients. Preoperative biliary drainage (PBD) was conducted in the following situations: hyperbilirubinemia with a total bilirubin (TB) level ≥ 15 mg/dl (> 258 μmol/L), preoperative cholangitis occurred, poor nutritional status before operation when needed nutritional support [10]. Prophylactic antibiotics were intravenously administered for 3 days (the operation day and postoperative day 1 and 2).

Drain amylase concentration, bacterial smear, and culture were conducted on postoperative day (POD) 1,3,5,7 to detect pancreatic fistula and intra-abdominal infection. The peritoneal drainage tubes removed on POD 7 after the abdominal enhanced computed tomography (CT) conducted on POD 7 showed no evidences of CR-POPF or fluid collection were found.

### Clinical data collection and definition of complication

Clinical data, including demographics (age, sex, hypertension, diabetic mellitus, BMI, preoperative jaundice, PBD), preoperative laboratory data (alanine aminotransferase, aspartate aminotransferase, alkaline phosphate, γ-glutamyl transferase, total bilirubin, direct bilirubin, albumin, white blood cell, hemoglobin, platelet), intraoperative variables (surgical method, vessel resection, operating time, volume of blood loss and transfusion, pancreatic texture and diameter of the main pancreatic duct), the fistula risk score (FRS) [11] and pathological diagnosis. Clavien–Dindo classification was applied for postoperative complications, with major complications defined as grade ≥ III [12]. CR-POPF (Grade B/ C), biliary leakage (BL), chylous fistula, delayed gastric emptying (DGE), and post-pancreatectomy hemorrhage (PPH) were diagnosed according to the International Study Group of Pancreatic Surgery (ISGPS) [13–16]. Wound infection, intra-abdominal infection, bacteremia, pneumonia, urinary tract infection were all included.

### Statistical analysis

Clinical data was analyzed using SPSS 23.0 software for Windows (SPSS Inc.). Categorical variables were compared with ^*2*^ test or Fisher’s exact test, with absolute number and percentage expressed. Continuous variables were analyzed by independent *t-*test, with mean and standard deviation (SD) expressed when the data showed normal distribution. Mann–Whitney *U* test was applied and showed as median (interquartile range, IQR) when they were not normally distributed. Univariate and multivariate logistic regression analysis of CR-POPF were completed using all patients enrolled. All variables with P < 0.1 in univariate analysis entered the multivariate logistic regression model with a stepwise forward approach to find out the independent risk factors for CR-POPF. Odds ratio (OR) and 95% confidence intervals (95%CI) were obtained. *P* < 0.05 was considered as statistics significantly.

A 1:1 nearest-neighbor propensity score-matching (PSM) analysis was performed to compare VAC group and none-VAC group adjusting preoperative jaundice, preoperative biliary drainage, alanine aminotransferase, aspartate aminotransferase, alkaline phosphate, γ-glutamyl transferase, total bilirubin, direct bilirubin, albumin, hemoglobin, platelet, surgical method, vessel resection, pancreatic texture, pancreatic duct diameter, intraoperative of blood loss and fistula risk score. Caliper matching on propensity score was estimated, and pairs were matched to within a range of 0.2 standard deviation of the logistic model of the propensity score.

## Results

### Patient characteristics

In our study, 263 patients were included during the 2-year study period. They were classified into two groups as VAC group and non-VAC group according to the pathological result of the specimen. The clinicopathological variables of all patients were shown in Table 1. There were 94 (35.7%) patients diagnosed with VAC, 61 (23.2%) with pancreatic ductal carcinoma, 24 (9.1%) with distal cholangiocarcinoma, 45 with pancreatic cystic neoplasms (25 IPMN, 10 SCN, 4MCN and 6 SPN), 9 with pNET, 4 with chronic pancreatitis and 26 other types. Furthermore, there were 163 (62%) males and the mean age of the entire cohort was 61.5 ± 12.1 years. Before surgery, levels of total bilirubin (TB) and direct bilirubin (DB) were 16.0 (9.2–58.6) μmol/L and 6.5 (2.3–45.2) μmol/L, respectively. A total of 103 (39.2%) were diagnosed with preoperative jaundice and 77 (29.3%) performed PBD. 99 (37.6%) patients developed CR-POPF, 106 (40.3%) patients underwent intra-abdominal infection, and 57 (21.7%) patients developed major complications (Calvin-Dindo grade ≥  III).

**Table 1:** Clinical characteristic of all patients

Characteristic	Total(n = 263)
Age (mean ± SD), years	61.5 ± 12.1
Sex, n (%)	
Male	163 (62.0)
Female	100 (38.0)
BMI (mean ± SD), kg/m^2^	23.5 ± 3.3
Diabetic mellitus, n (%)	47 (17.9)
Hypertension, n (%)	89 (33.8)
History of surgery, n (%)	79 (30.0)
Smoking, n (%)	61 (23.2)
Alcohol, n (%)	77 (29.3)
Preoperative jaundice, n (%)	103 (39.2)
Preoperative biliary drainage, n (%)	77 (29.3)
ALT (median, IQR), U/L	45.5 (17.8–102.8)
AST (median, IQR), U/L	30.3 (17.9–63.5)
AKP (median, IQR), U/L	141.2 (69.5–290.2)
γ-GGT (median, IQR), U/L	130.6 (22.9–387.8)
TB (median, IQR), μmol/L	16.0 (9.2–58.6)
DB (median, IQR), μmol/L	6.5 (2.3–45.2)
Albumin (mean ± SD), g/L	38.8 ± 3.1
WBC(mean ± SD), × 10^9^/L	5.8 ± 1.8
Hemoglobin (mean ± SD), g/L	122.8 ± 17.9
Platelet(mean ± SD), × 10^9^/L	230.9 ± 86.8
Pathology diagnosis, n (%)	
VAC	94 (35.7)
PDAC	61 (23.2)
DCC	24 (9.1%)
IPMN	25 (9.5%)
SCN	10 (3.8%)
pNET	9 (3.4%)
SPN	6 (2.3%)
MCN	4 (1.5%)
CP	4 (1.5%)
Others	26 (9.9%)
Surgical method, n (%)	
PD	186 (70.7)
PPPD	77 (29.3)
Vessel resection, n (%)	
Yes	9 (3.4)
No	254 (96.6)
Pancreatic texture	
Firm	36 (13.7)
Soft	227 (86.3)
Diameter of the MPD (mm)	3.0 (2.0–5.0)
Fistula risk score	6.0 (4.0–7.0)
Operating time (mean ± SD), min	379.4 ± 96.9
Blood loss volume (median, IQR), ml	400.0 (300.0–700.0)
Blood transfusion (median, IQR), ml	0.0 (0.0–700.0)
CR-POPF, n (%)	99 (37.6)
Biliary leakage, n (%)	16 (6.1)
DGE, n (%)	94 (35.7)
PPH, n (%)	22 (8.4)
Chylous fistula, n (%)	31 (11.8)
Major postoperative complications, n (%)	57 (21.7)
Wound infection, n (%)	14 (5.3)
Intra-abdominal infection, n (%)	106 (40.3)
Bacteremia, n (%)	14 (5.3)
Pneumonia, n (%)	8 (3.0)
Urinary tract infection, n (%)	3 (1.4)

SD: standard deviation; IQR: interquartile; BMI: body mass index; ALT: alanine aminotransferase, AST: aspartate aminotransferase, AKP: alkaline phosphate, γ-GGT:γ-glutamyl transferase, TB:total bilirubin, DB: direct bilirubin; WBC: white blood cell; VAC: Vater’s ampullary carcinoma; PDAC: pancreatic ductal adenocarcinoma; DCC: distal cholangiocarcinoma; IPMN: intraductal papillary mucinous neoplasm; SPN: solid pseudopapillary neoplasm of the pancreas; SCN: pancreatic serous cystadenoma; MCN: mucinous cystadenoma of pancreas; pNET: pancreatic neuroendocrine tumor; CP: chronic pancreatitis; MPD: main pancreatic duct; PD: pancreaticoduodenectomy; PPPD: pylorus-preserving pancreaticoduodenectomy; CR-POPF: Clinically relevant postoperative pancreatic fistula (Grade B/ C), PPH: post-pancreatectomy hemorrhage; DGE: delayed gastric emptying

### Risk factors for CR-POPF

In univariate analysis, age (OR = 1.031, 95% CI = 1.003–1.060, *P* = 0.030), pathology (VAC vs. non-VAC) (OR = 2.423, 95% CI = 1.223–4.800, *P* = 0.011), and surgical method (PD vs. PPPD) (OR = 0.496, 95% CI = 0.253–0.973, *P* = 0.041) were significantly associated with CR-POPF. In multivariate analysis, only pathology (VAC vs. non-VAC) (OR = 1.824, 95% CI = 1.087–3.060 *P* = 0.023) was the independent risk factor of CR-POPF (Table 2).

**Table 2:** Risk factors of CR-POPF: Univariate and multivariate logistic regression analysis

Variables	Univariate analysis		Multivariate analysis	
	OR (95%CI)	*P *value	OR (95%CI)	*P *value
Age	1.031 (1.003–1.060)	0.030	NA	NA
Sex, male vs. female	0.677 (0.348–1.317)	0.251		
BMI	1.041 (0.948–1.142)	0.399		
Diabetic mellitus	1.122 (0.531–2.372)	0.763		
Hypertension	1.031 (0.545–1.950)	0.926		
History of surgery	0.523 (0.271–1.012)	0.054	NA	NA
Smoking	0.455 (0.180–1.153)	0.097	NA	NA
Alcohol	0.835 (0.310–2.248)	0.721		
Preoperative jaundice, yes vs. no	1.949 (0.725–5.242)	0.186		
Preoperative biliary drainage, yes vs. no	0.961 (0.371–2.489)	0.934		
ALT	0.999 (0.992–1.007)	0.862		
AST	0.998 (0.989–1.008)	0.719		
AKP	0.998 (0.996–1.001)	0.136		
γ-GGT	1.000 (0.999–1.002)	0.473		
TB	0.983 (0.951–1.017)	0.321		
DB	1.024 (0.979–1.072)	0296		
Albumin	1.101 (0.971–1.249)	0.134		
WBC	1.157 (0.969–1.382)	0.107		
Hemoglobin	1.014 (0.992–1.036)	0.217		
Platelet	1.002 (0.998–1.006)	0.425		
Pathology, VAC vs. non-VAC	2.423 (1.223–4.800)	0.011	1.824 (1.087–3.060)	0.023
Surgical method, PD vs. PPPD	0.496 (0.253–0.973)	0.041	NA	NA
Vessel resection, yes vs. no	1.051 (0.186–5.940)	0.955		
Operating time	1.003 (0.999–1.006)	0.153		
Blood loss volume	0.999 (0.998–1.000)	0.207		
Blood transfusion	1.000 (1.000–1.001)	0.311		

BMI: body mass index; ALT: alanine aminotransferase, AST: aspartate aminotransferase, AKP: alkaline phosphate, γ-GGT:γ-glutamyl transferase, TB: total bilirubin, DB: direct bilirubin, WBC: white blood cell, NA: not applicable, VAC: Vater’s ampullary carcinoma; PD: pancreaticoduodenectomy; PPPD: pylorus-preserving pancreaticoduodenectomy; CR-POPF: Clinically relevant postoperative pancreatic fistula (Grade B/ C); CI: confidence interval; OR: odds ratio

### Propensity score-matched analysis

As shown in Table 3, patients diagnosed VAC had higher level of platelet and FRS, lower level of hemoglobin and albumin and smaller main pancreatic duct dimeter. At the same time, patients in the VAC group had higher level of alanine aminotransferase (ALT), aspartate aminotransferase (AST), alkaline phosphate (AKP), γ-glutamyl transferase(γ-GGT), total bilirubin (TB), and direct bilirubin (DB), indicated these patients may have worse liver function. Furthermore, the rate of preoperative jaundice, biliary drainage and pancreatic texture showed statistical difference.

**Table 3:** Baseline characteristics in the unmatched and matched group according to the pathological diagnosis

Variables	Before PS matching			After PS matching		
	Non-VAC (n = 169)	VAC (n = 94)	*P *value	Non-VAC (n = 59)	VAC (n = 59)	*P *value
Age (mean ± SD), years	60.9 ± 12.5	62.7 ± 11.4	0.251	60.5 ± 13.6	62.9 ± 10.8	0.306
Sex, n (%)			0.945			0.450
Male	105 (62.1)	58 (61.7)		34 (57.6)	38 (64.4)	
Female	64 (37.9)	36 (38.3)		25 (42.4)	21 (35.6)	
BMI (mean ± SD), kg/m^2^	23.4 ± 3.1	23.7 ± 3.9	0.591	22.8 ± 2.8	24.0 ± 3.4	0.060
Diabetic mellitus, n (%)	34 20.1)	13 (13.8)	0.202	14 (23.7)	10 (16.9)	0.360
Hypertension, n (%)	58 (34.3)	31 (32.9)	0.826	23 (38.9)	21 (35.6)	0.703
History of surgery, n (%)	50 (29.5)	29 (30.8)	0.830	18 (30.5)	20 (33.9)	0.694
Smoking, n (%)	38 (22.4)	23 (24.4)	0.715	12 (20.3)	14 (23.7)	0.657
Alcohol, n (%)	33 (19.5)	13 (13.8)	0.244	10 (16.9)	8 (13.6)	0.609
Preoperative jaundice, n (%)	52 (30.77)	51 (54.26)	< 0.001	31 (52.5)	29 (49.2)	0.713
Preoperative biliary drainage, n (%)	32 (18.93)	45 (47.87)	< 0.001	23 (38.9)	23 (38.9)	1.000
ALT (median, IQR), U/L	32.8 (14.9–74.8)	77.1 (30.5–134.4)	< 0.001	62.7 (29.0–85.6)	74.8 (25.9–143.7)	0.505
AST (median, IQR), U/L	27.3 (16.3–49.8)	44.4 (22.8–100.1)	< 0.001	36.5 (22.8–69.6)	44.5 (20.3–95.0)	0.400
AKP (median, IQR), U/L	87.6 (64.3–244.8)	223.2 (122.6–369.5)	< 0.001	219.7 (74.0–351.5)	192.2 (91.9–317.3)	0.921
γ-GGT(median, IQR), U/L	45.2 (19.3–298.4)	270.8 (98.9–562.7)	< 0.001	193.3 (30.1–497.3)	215.2 (58.5–558.4)	0.669
TB (median, IQR), U/L	13.5 (8.9–56.4)	38.5 (11.5–66.3)	0.023	47.2 (9.7–105.9)	33.8 (9.4–78.1)	0.394
DB (median, IQR), U/L	4.0 (2.1–42.0)	27.5 (5.3–46.2)	0.002	34.9 (2.7–79.9)	23.6 (2.4–57.8)	0.427
Albumin (mean ± SD), g/L	39.4 ± 3.2	37.6 ± 2.6	< 0.001	37.6 ± 3.1	38.1 ± 2.3	0.402
WBC(mean ± SD), × 10^9^/L	5.7 ± 1.6	5.9 ± 2.0	0.422	6.2 ± 1.9	5.9 ± 2.1	0.378
Hemoglobin (mean ± SD), g/L	125.9 ± 16.8	117.1 ± 18.6	< 0.001	117.7 ± 15.3	121.1 ± 16.1	0.245
Platelet(mean ± SD), × 10^9^/L	214.8 ± 78.6	260.0 ± 93.7	< 0.001	248.1 ± 95.5	233.9 ± 70.2	0.363
Surgical method, n (%)			0.017			0.059
PD	128 (75.7)	58 (61.7)		45 (76.3)	35 (59.3)	
PPPD	41 (24.3)	36 (38.3)		14 (23.7)	24 (40.7)	
Vessel resection, n (%)			0.023			NA
No	160 (94.7)	94 (100.0)		0 (0.0)	0 (0.0)	
Yes	9 (5.3)	0 (0.0)		59 (100.0)	59 (100.0)	
Pancreatic texture			0.010			0.569
Firm	30 (17.8)	6 (6.4)		8 (13.6)	6 (10.2)	
Soft	139 (82.2)	88 (93.6)		51 (86.44)	53 (89.83)	
Diameter of the MPD (mm)	3.0 (2.0–5.0)	2.0 (2.0–4.0)	0.003	3.0 (2.0–4.0)	3.0 (2.0–5.0)	0.536
Fistula risk score	5.0 (3.0–7.0)	6.0 (5.0–7.0)	0.001	6.0 (4.0–7.0)	6 (4.0–7.0)	0.434
Operating time (mean ± SD), min	382.6 ± 105.1	373.7 ± 80.6	0.446	376.5 ± 86.9	388.5 ± 75.1	0.426
Blood loss volume (median, IQR), ml	500.0 (300.0–700.0)	400.0 (300.0–600.0)	0.069	400.0 (300.0–500.0)	400 (300.0–600.0)	0.678
Blood transfusion (median, IQR), days	0.0 (0.0–737.5)	0.0 (0.0–600.0)	0.524	0.0 (0.0–750.0)	0.0 (0.0–600.0)	0.431

SD: standard deviation; IQR: interquartile; BMI: body mass index; PS: propensity score; NA: not applicable; ALT: alanine aminotransferase, AST: aspartate aminotransferase, AKP: alkaline phosphate, γ-GGT:γ-glutamyl transferase, TB: total bilirubin, DB: direct bilirubin, albumin; WBC: white blood cell; VAC: Vater’s ampullary carcinoma; PD: pancreaticoduodenectomy; PPPD: pylorus-preserving pancreaticoduodenectomy, MPD: main pancreatic duct

In order to adjust the differences of baseline variables in each group, a 1:1 nearest-neighbor propensity score matching (PSM) analysis was conducted. After PSM, a balanced cohort included the VAC group as observational group (59 patients) and the non-VAC group as the control group (59 patients). All baseline characteristics were comparable after PSM.

### Postoperative complications

After PSM, CR-POPF occurred in 28 (47.5) patients in the VAC group and 16 (27.1%) patients in the non-VAC group (*P* = 0.025; Table 4). Both before and after PSM, the intra-abdominal infection occurred more frequently in the VAC group significantly. While major postoperative complication rates had a higher tendency in patients diagnosed with VAC before PSM, the differences were not statistically significant both before and after PSM. Furthermore, the rates of biliary leakage, chylous fistula, delayed gastric emptying (DGE), post-pancreatectomy hemorrhage (PPH), wound infections, bacteremia, pneumonia, and urinary tract infection were comparable between the two groups.

**Table 4:** Postoperative mortality and morbidity according to the pathological diagnosis

Variables	Before PS matching			After PS matching		
	VAC (n = 94), n (%)	Non-VAC (n = 169), n (%)	*P *value	VAC (n = 59), n (%)	non-VAC (n = 59), n (%)	*P *value
CR-POPF, n (%)	44 (46.8)	55 (32.5)	0.022	28 (47.5)	16 (27.1)	0.025
Biliary leakage, n (%)	6 (6.3)	10 (5.9)	0.880	4 (6.8)	3 (5.1)	0.697
Chylous fistula, n (%)	12 (12.7)	19 (11.2)	0.713	10 (16.9)	9 (15.3)	0.802
DGE, n (%)	35 (37.2)	59 (34.9)	0.706	20 (33.9)	21 (35.6)	0.847
PPH, n (%)	8 (8.5)	14 (8.3)	0.949	5 (8.5)	3 (5.1)	0.464
Major postoperative complications, n (%)	26 (27.6)	31 (18.3)	0.079	13 (22.0)	7 (11.9)	0.141
Wound infection, n (%)	6 (6.3)	8 (4.7)	0.723	5 (8.5)	2 (3.4)	0.242
Intra-abdominal infection, n (%)	49 (52.1)	57 (33.7)	0.004	30 (50.9)	17 (28.8)	0.015
Bacteremia, n (%)	6 (6.3)	8 (4.7)	0.568	2 (3.4)	3 (5.1)	0.648
Pneumonia, n (%)	1 (1.1)	7 (4.1)	0.164	1 (1.7)	2 (3.4)	0.559
Urinary tract infection, n (%)	1 (1.1)	2 (1.2)	0.930	1 (1.7)	1 (1.7)	1.000

VAC: Vater’s ampullary carcinoma; CR-POPF: clinically relevant postoperative pancreatic fistula (Grade B/ C); PPH: post-pancreatectomy hemorrhage; DGE: delayed gastric emptying; PS: propensity score

## Discussion

In our study, the rate of CR-POPF, major postoperative complication were 37.6% and 21.7%, respectively, and consistent with previous studies [17–22]. We also identified that pathology diagnose (VAC vs. non-VAC) was the independent risk factor for CR-POPF. Furthermore, in order to reduce the bias of baseline variables between two groups, we performed an additional analysis by propensity score-matching (PSM). The occurrence of CR-POPF and intra-abdominal infection between the two groups showed statistically difference both before and after PSM.

The most hazardous postoperative complication of PD is CR-POPF, which is the greatest contributor to postoperative morbidity and mortality after PD as reported previously [23–25]. Numerous independent risk factors of CR-POPF now are identified. These include “patient-related factors” such as age, BMI, and hemoglobin, “pancreatic factors” such as pancreatic texture and diameter of MPD, “surgical factors” such as intraoperative blood lose and vessel resection [10, 26, 27]. In previous studies, pancreatic ductal adenocarcinoma was a protective factor of the development of CR-POPF [11, 28]. However, the impact of Vater’s ampullary carcinoma on postoperative complications, especially CR-POPF has yet to be adequately researched.

In the univariate analysis of our study, VAC patients were more likely to have an increased rate of preoperative jaundice and preoperative biliary drainage. Moreover, the preoperative serum level of transaminase and bilirubin, signs of liver function, were statistically higher in the VAC group. Patients with VAC had significantly higher CR-POPF rate compared with patients with other diseases (46.8% vs. 32.5%, *P* = 0.022). On one hand, jaundice or high level of preoperative serum bilirubin may lead to high levels of serum proinflammatory cytokines and endotoxin, which have been proved in several animal models [29, 30]. On the other hand, intestinal mucosal barrier function was interfered by obstructive jaundice, which accelerates bacterial translocation [31, 32]. At the same time, impaired hepatocellular function, leads to insufficient protein synthesis, and lower hemoglobin level delayed healing of wound especially the pancreatojejunostomy that threaten patients’ recovery [33–35]. Part of patients’ primary complaint is gastrointestinal bleed and melena because part of the lesions of ampullary cancer showed ulcerative type. That may explain why patients in the VAC-group had significantly lower hemoglobin level than the patients in non-VAC group in our study. At the same time, the ulcerative type of VAC and anemia may result in translocation and invasion of intestinal bacteria, which lead to the development of CR-POPF and increase the susceptibility to infections [9, 33].

Numerous studies, including prospective studies and random controlled trails, have been performed for the negative impact of PBD on surgical outcomes. Most studies among them demonstrated that biliary drainage increased the rate of postoperative complications including CR-POPF [36–39]. A high-quality RCT article identified that PBD increase the risk of postoperative complication for cancer of the pancreatic head [40].

PSM was conducted to further analyze the postoperative outcomes in VAC group and non-VAC group, higher CR-POPF, PPH, intra-abdominal infection rates were observed in VAC patients both before and after accounting for other variables. Another mechanism by which VAC increase the risk of CR-POPF may relate to the effect of softer pancreas texture. Periampullary lesions except PDAC and chronic pancreatitis always do not cause fibrotic reactions. As a result, performing pancreatojejunostomy after the resection of VAC is expected to be more challenging induced by the soft pancreatic texture, which increase the risk of anastomotic leakage. Kawai et al. identified that a soft pancreas was a significant risk factor of CR-POPF from 11 Japanese medical centers [41]. Callery et al. [11] conducted a validated pancreatic fistula risk score and four risk factors are identified including excessive blood lose, softer pancreatic gland, smaller pancreatic duct and high-risk pathologies such as duodenal and ampullary tumors. These results were in line with our studies.

As our best knowledge, the present study is the first retrospective cohort focused on the impact of pathology VAC about the postoperative complications especially CR-POPF. Although selection bias cannot be totally eliminated, we performed PSM to adjust the baseline variables and reduce the bias. After PSM, postoperative complications can be compared between the two groups. The result of our studies demonstrated that VAC increases the development of CR-POPF, PPH and intra-abdominal infection. Although it is difficult to evaluate the texture of pancreas before surgery accurately, the possibility of CR-POPF even postoperative complications could be assessed by guessing the pathology through preoperative CT or endoscopic biopsy.

The present study has some limitations. First, it was a retrospective study with a selection and historical background bias. Second, the study was a small study from a single center, to validate the impact of pathology VAC on CR-POPF even postoperative complications, future randomize controlled trails are indispensable.

## Conclusions

VAC was an independent risk factor of CR-POPF after PD. At the same time, the VAC patients experience a higher rate of PPH and intra-abdominal infection even after PSM. Therefore, these observations strongly support a cautious evaluation before PD and better perioperative management for patients diagnosed VAC.

## Data Availability

All data generated or analyzed during this study are available from the corresponding author on reasonable request.

## Abbreviations

PD: Pancreaticoduodenectomy
CR-POPF: Clinically relevant postoperative pancreatic fistula
PPH: Post-pancreatectomy hemorrhage
MPD: Main pancreatic duct
PF: Pancreatic fistula
PDAC: Pancreatic ductal adenocarcinoma
DCC: Distal cholangiocarcinoma
VAC: Vater’s ampullary carcinoma
PBD: Preoperative biliary drainage
TB: Total bilirubin
POD: Postoperative day
DGE: Delayed gastric emptying
ISGPS: The International Study Group of Pancreatic Surgery
OR: Odds ratio
CI: Confidence intervals
PSM: Propensity score-matching
